# Safety monitoring in inactivated COVID-19 vaccines by clinical pharmacists from a single center in China

**DOI:** 10.3389/fimmu.2022.882919

**Published:** 2022-09-05

**Authors:** Min Hu, Wei Guo, Li Liu, Yu Yang, Qiling Xu, Fang Cheng, Fang Zeng, Yu Zhang

**Affiliations:** ^1^ Department of Pharmacy, Union Hospital, Tongji Medical College, Huazhong University of Science and Technology, Wuhan, China; ^2^ Hubei Province Clinical Research Center for Precision Medicine for Critical Illness, Wuhan, China

**Keywords:** COVID-19, SARS-CoV-2, inactivated vaccine, safety, adverse events

## Abstract

Given that vaccine-induced adverse effects were mostly based on previous laboratory research and clinical trials, real-world data on the safety of coronavirus disease 2019 (COVID-19) vaccination were lacking. This study reported the adverse events (AEs) among inactivated COVID-19 vaccine recipients. Data were collected from a total of 2,808 hospital employees and their family members in Wuhan, China, with all of them receiving the first dose of inactivated COVID-19 vaccines from two pharmaceutical companies. The first dose was given between 29th April and 13th May 2021. A total of 2,732 vaccinees received the second dose between 27th May and 8th July 2021. The whole process of receiving the vaccine was monitored by clinical pharmacists, and the information on AEs including demographics, occurrence, types, and severity was recorded through an online questionnaire and telephone follow-up. Most of the common AEs were mild and tolerable, and the overall incidence of AEs was lower than the data from the safety profile in clinical trials. Moreover, the incidence of AEs in the first dose (21.30%, 598) was higher than that in the second dose (16.07%, 439). Furthermore, the first injection had more severe AEs (4, 0.14%) than the second injection (2, 0.07%). The AEs involved the skin, muscle, respiratory tract, gastrointestinal tract, cardiovascular system, and other tissues and systems. The most common AE was pain at the injection site (first dose: 10.19%, second dose: 12.55%). All the vaccinees with AEs for both doses recovered fully in the end. It was noted that some AEs might cause blood coagulation disorder and bleeding risk. Therefore, ongoing monitoring of AEs after COVID-19 vaccination is essential in evaluating the benefits and risks of each vaccine.

## Introduction

As a highly transmissible and pathogenic coronavirus that emerged in late 2019, severe acute respiratory syndrome coronavirus 2 (SARS-CoV-2) has caused a pandemic of acute respiratory disease, named “coronavirus disease 2019” (COVID-19), and has infected a significant portion of the world’s population (1). During the COVID-19 pandemic, people across the globe have been facing major healthcare challenges, lockdowns, stress, and anxiety, since there has been no specific treatment for this pandemic. Given the lack of specific therapy and the rapid spread of this virus, vaccination would play an essential role in fighting against the COVID-19 pandemic (2). Vaccines against COVID-19 are being developed at an unprecedented speed. More recently, more than 1.5 billion doses of the COVID-19 vaccine have been administered globally, with China ranking first.

The World Health Organization (WHO) has approved two of China’s COVID-19 vaccines for use worldwide in 2021. However, the published trial data remain scarce (3). The challenges for postmarketing safety monitoring have been put forward (4). Even for relatively mature technology platforms like inactivated vaccines and recombinant protein-based vaccines, the specifications for the vaccines and methods applied by different enterprises are difficult to standardize and unify for comparison (5). This could be partly attributed to the urgent need and poor knowledge based on research for rolling out a vaccine. How to timely update and improve the relevant specifications and guidelines associated with the safety evaluation of vaccines based on new data from the real world becomes urgent.

Two SARS-CoV-2 inactivated vaccines (referred to as WIV04 and HB02 vaccines hereafter) used in China were designed by the Wuhan Institute of Biological Products Co., Ltd. and the Beijing Institute of Biological Products Co., Ltd., both of which belong to the China National Biotec Group Company Limited (6). Unlike Western countries, vaccines in China are provided by production enterprises and distributed by the Centers for Disease Control (CDC) or CDC-assigned health stations and later given out by qualified vaccinators, and most of the time, the hospitals are not involved. Due to the emergency use of COVID-19 vaccines, healthcare professionals were the first group to receive the vaccines. The adverse events (AEs) of these inactivated vaccines were noted. Two inactivated vaccines against COVID-19 have been shown to be generally safe and effective in adults in phase I/II trials (7), which was also confirmed by the data from phase III clinical trials. Seven days after each injection, AEs occurred in 41.7% to 46.5% of the participants in the two groups (6). However, since the postmarketing data for the two vaccines have not been entirely acquired yet, it is essential to further confirm their safety in the real world. In the current study, the AEs after COVID-19 vaccination among the employees and their family members from the Union Hospital, Tongji Medical College, Huazhong University of Science and Technology from 29th April to 13th May 2021 and from 27th May to 8th July 2021 were collected, and the safety of vaccination was estimated. These data provide reassurance and helpful information, which healthcare providers and vaccinees might expect after vaccination.

## Materials and methods

### Study design and population

In our study, the employees and their family members from the Union Hospital, Tongji Medical College, Huazhong University of Science and Technology in Wuhan, China, received the two inactivated COVID-19 vaccines (WIV04 and HB02 vaccines). Those who received the vaccine had no allergic diseases (such as asthma, allergic rhinitis, urticaria) and no history of allergies to the vaccine in addition to food or drug allergy. Two doses with an interval of 4–8 weeks were required. The vaccine was injected intramuscularly into the deltoid muscle, and a 30-min observation was strictly required after both doses of vaccination. AEs that presented 30 min after both doses were given were recorded on the spot by clinical pharmacists at the Union Hospital. Moreover, after that, the clinical pharmacists collected the required information using a structured pretested questionnaire through an online questionnaire and telephone follow-up, and AEs were monitored for 7 days.

Consent from the subjects was obtained according to the Declaration of Helsinki, and the study was approved by the ethics committee of the institution where the work was performed. The pretested questionnaire was formulated based on the objectives and was intended as a guide to obtain the necessary information from the receivers. The questionnaire encompassed 19 questions (closed- and open-ended questions) and is shown in **Supplementary Table S1**.

Vaccinee information contained demographic data and past medical and allergy histories. AE information contained the vaccination date, symptom onset date and time, area where the AE occurred, and prognosis. Two pharmacists with more than 7 years of experience independently analyzed the data, and if their classification results were different, a professor of pharmacists with more than 20 years of experience was sought to resolve the disagreement.

### Statistical analysis

The primary outcome of the survey was AEs after COVID-19 vaccination. The safety analyses included all vaccinees and whether they received one or two doses of the vaccine. Safety analyses were expressed as counts and percentages for solicited and unsolicited local reactions and systemic events during the period. Multinomial logistic regression is the extension of (binary) logistic regression when the categorical dependent outcome has more than two levels. This model was then developed to identify the factors associated with AEs, and the odds ratio (OR) and 95% confidence interval (CI) were calculated. Variables that were significant at the *P <*0.05 level in the univariate analyses were included in the model. All data were analyzed using IBM SPSS statistics 22.0 software. A *P*-value of <0.05 was considered to represent a statistically significant difference among the test populations.

## Results

### Demographic analysis and clinical information

From 29th April to 13th May 2021, the first dose of inactivated COVID-19 vaccines was injected into 2,808 hospital employees and their family members. Due to the AEs of the first dose, 2,732 of the abovementioned vaccinees participated in the second dose from 27th May to 8th July 2021. We summarized the clinical characteristics of the vaccinees with AEs in **Table 1**. The majority of vaccinees with AEs of both doses were women (72.24% for the first dose and 72.44% for the second dose). The body mass index (BMI) of the vaccinees with AEs for both doses was around 22. Furthermore, vaccinees who had a history of previous allergies (e.g., food, medicine, or vaccine) in dose 1 were more common (16.22%) than those in dose 2 (12.53%). Fifty-four (9.03%) of the vaccinees had medical histories in dose 1, while 38 (8.66%) of the vaccinees had medical histories in dose 2. Of note, the first injection had more AEs (598, 21.30%) than the second injection (439, 16.07%). Moreover, the first injection had more severe AEs (4, 0.14%) than the second injection (2, 0.07%). In dose 1, most of the AEs occurred more than 48 h after inoculation (39.97%) and lasted 30 min–24 h, while in dose 2, most of the AEs occurred within 30 min–24 h after inoculation (75.39%) and lasted 24–48 h. All the vaccinees with AEs for both doses recovered fully in the end.

**Table 1:** Baseline characteristics of the registry population with AEs.

Characteristic	First-dose vaccination	Second-dose vaccination	*P*-value
**Proportion of receivers with AEs**	598/2,808 (21.30%)	439/2,732 (16.07%)	<0.001
**Age (mean ± SD)**	40.14 ± 11.97	41.81 ± 13.64	0.78
**Sex**			
Male	166 (27.76%)	121 (27.56%)	0.56
Female	432 (72.24%)	318 (72.44%)	0.59
**Height (mean ± SD)**	164.31 ± 7.40	163.70 ± 7.38	0.62
**Weight (mean ± SD)**	60.75 ± 11.35	60.40 ± 12.38	0.54
**BMI (mean ± SD)**	22.36 ± 2.84	22.42 ± 3.52	0.56
**Allergy histories**	97 (16.22%)	55 (12.53%)	0.003
**Medical histories**	54 (9.03%)	38 (8.66%)	0.041
**Occupation**			
Doctor	146 (24.41%)	107 (24.37%)	0.54
Nurse	156 (26.09%)	97 (22.10%)	0.15
Technician	62 (10.37%)	31 (7.06%)	0.12
Administrator	3 (0.5%)	14 (3.19%)	0.02
Researcher	11 (1.84%)	6 (1.37%)	0.72
Cleaner	7 (1.17%)	7 (1.59%)	0.65
Other^a^	213 (35.62%)	177 (40.32%)	0.005
**Severity**			
Severe	4 (0.14%)	2 (0.07%)	0.008
Common	594 (21.15%)	437 (16.00%)	<0.001
**Time the adverse reactions occurred**			
Within 5 min after inoculation	54 (9.03%)	36 (8.20%)	0.67
Within 5–30 min after inoculation	31 (5.18%)	13 (2.96%)	0.058
Within 30 min–24 h after inoculation	237 (39.63%)	331 (75.39%)	<0.001
Within 24 to 48 h after inoculation	37 (6.19%)	36 (8.20%)	0.49
>48 h after inoculation	239 (39.97%)	23 (5.24%)	<0.001
**Duration of the adverse reactions**			
Less than 30 min	17 (2.84%)	5 (1.39%)	0.06
30 min–24 h	195 (32.61%)	99 (22.55%)	0.02
24–48 h	122 (20.4%)	151 (34.40%)	0.04
48–72 h	121 (20.23%)	93 (21.18%)	0.58
More than 72 h	136 (22.74%)	91 (20.73%)	0.64
**The ending (healed)**	598 (100%)	439 (100%)	0.5

P < 0.001: second-dose vaccination vs. first-dose vaccination.

a Registry population other than doctor, nurse, technician, administrator, researcher, or cleaner.

### AE classifications

**Figure 1:**
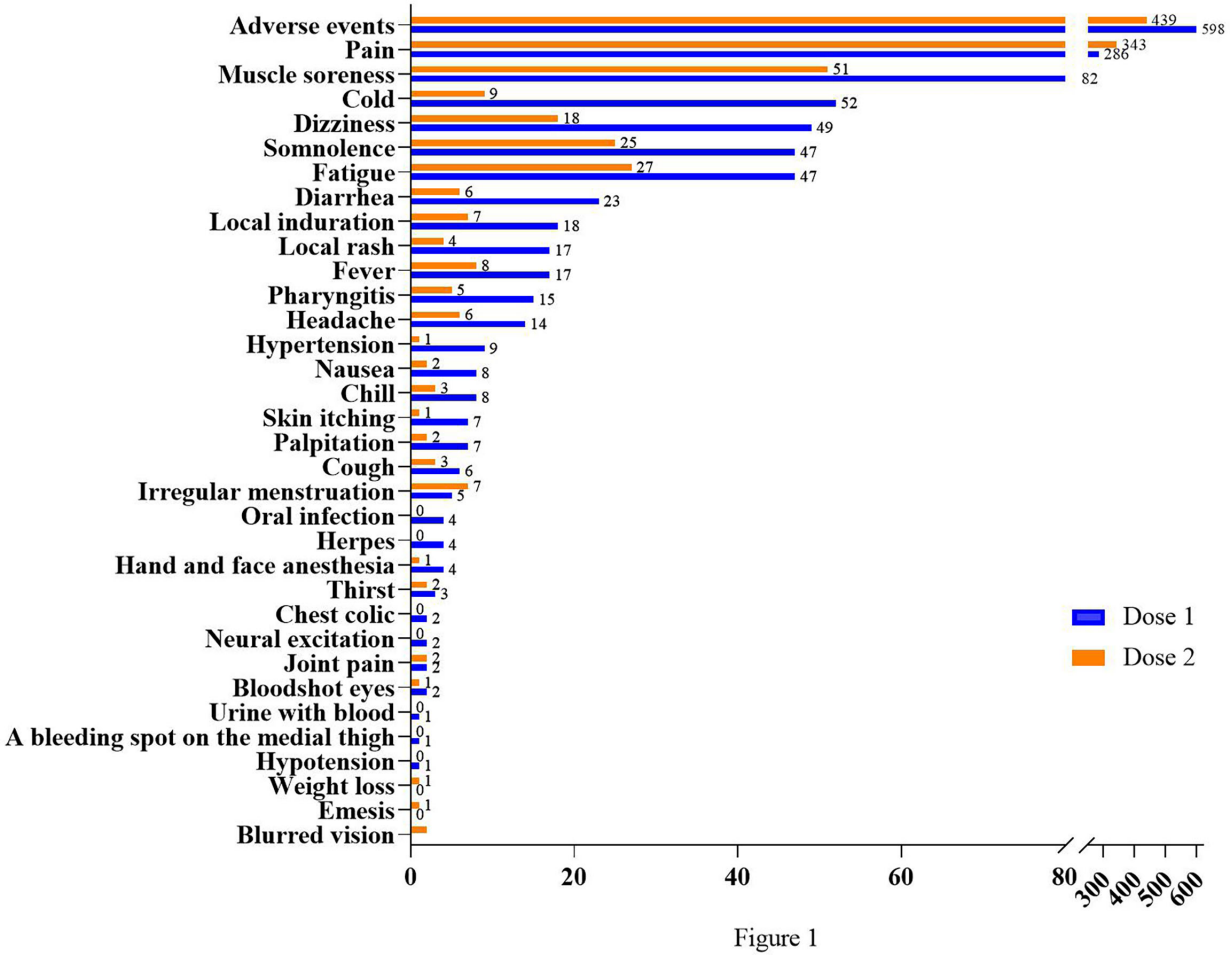
Comparison between frequencies of common adverse effects after two doses. The abscissa shows the number of cases; the ordinate shows different kinds of AEs.

The inactivated COVID-19 vaccines would carry common adverse effects after the first or the second dose. The various potential adverse effects of vaccination included in this study are presented in **Table 2**. A comparison between the occurrence rate of common adverse effects of inactivated COVID-19 vaccines for doses 1 and 2 is shown in **Figure 1**. For both doses, pain (10.19% for dose 1, 12.55% for dose 2) ranked as the highest frequent AE. Muscle soreness (2.92% for dose 1, 1.87% for dose 2) ranked as the second highest frequent AE. Moreover, cold-related symptoms such as cold, dizziness, somnolence, and fatigue also commonly occurred after the first vaccination. Among skin-related disorders, symptoms like local induration and local rash were most commonly reported. Gastrointestinal disorders such as diarrhea, nausea, and emesis were also included. The inactivated COVID-19 vaccines also affected the oral cavity, cardiovascular system, and nervous system. In particular, we identified some scarce but concerning coagulation function disorders, such as a bleeding spot on the medial thigh (0.04% for dose 1, 0.00% for dose 2), hematuresis (0.04% for dose 1, 0.00% for dose 2), irregular menstruation (0.18% for dose 1, 0.26% for dose 2), and bloodshot congested eyes (0.07% for dose 1, 0.11% for dose 2). Furthermore, it was indicated that gender could influence the AEs (**Supplementary Table S2**).

**Table 2:** Common manifestations of adverse events after vaccination.

Adverse event types	First-dose vaccination	Second-dose vaccination
**Pain**	286 (10.19%)	343 (12.55%)
**Cold-related symptoms**		
Cold	52 (1.85%)	9 (0.33%)
Headache	14 (0.50%)	6 (0.22%)
Dizziness	49 (1.75%)	18 (0.66%)
Fever	17 (0.61%)	8 (0.29%)
Somnolence	47 (1.67%)	25 (0.92%)
Fatigue	47 (1.67%)	27 (0.99%)
Chill	8 (0.28%)	3 (0.11%)
Cough	6 (0.21%)	3 (0.11%)
Pharyngitis	15 (0.53%)	5 (0.18%)
**Skin-related symptoms**		
Local induration	18 (0.64%)	7 (0.26%)
Local rash	17 (0.60%)	4 (0.15%)
Skin itching	7 (0.24%)	1 (0.04%)
Herpes	4 (0.14%)	0 (0.00%)
**Nerve**		
Neural excitation	2 (0.07%)	0 (0.00%)
Hand and face anesthesia	4 (0.14%)	1 (0.04%)
Blurred vision	0 (0.00%)	2 (0.07%)
**Muscle joints**		
Muscle soreness	82 (2.92%)	51 (1.87%)
Joint pain	2 (0.07%)	2 (0.07%)
**Oral cavity**		
Dry mouth	3 (0.11%)	2 (0.07%)
Oral infection	4 (0.14%)	0 (0.00%)
**Gastrointestinal tract**		
Diarrhea	23 (0.82%)	6 (0.22%)
Nausea	8 (0.28%)	2 (0.07%)
Emesis	0 (0.00%)	1 (0.04%)
**Cardiovascular**		
Chest colic	2 (0.07%)	0 (0.00%)
Palpitation	7 (0.25%)	2 (0.07%)
Lower blood pressure	1 (0.04%)	0 (0.00%)
Elevated blood pressure	9 (0.32%)	1 (0.04%)
**Coagulation function**		
A bleeding spot on the medial thigh	1 (0.04%)	0 (0.00%)
Hematuresis	1 (0.04%)	0 (0.00%)
Irregular menstruation	5 (0.18%)	7 (0.26%)
Bloodshot congested eyes	2 (0.07%)	3 (0.11%)

In general, the safety of the inactivated COVID-19 vaccines for both doses was good. Most of the common adverse reactions were mild and tolerable, with generally low incidence. Except for local pain, most AEs were more common with the first dose than the second dose.

### Pain evaluation after COVID-19 vaccination

The participants were evaluated with a visual analog scale (VAS). In total, there were 286 vaccinees with pain in dose 1 and 343 vaccinees with pain in dose 2, which were further evaluated. **Table 3** summarizes the characteristics of these receivers with pain and their feelings of pain. According to the survey, more people felt pain on the second dose than on the first dose. The majority of the participants had a pain score of 1 in the first dose, but most participants had a pain score of 2 in the second dose. For both doses, the time of pain mostly occurred within 30 min–24 h after inoculation and in the upper arm. In addition, there were other complications that occurred with pain, such as drowsiness, dizziness, fatigue, cold, fever, rash, palpitation, skin itching, local induration, muscle soreness, night sweat, and others. Of note, more than 79% of the recipients with pain had no complications, and the proportion of pain relieved after 7 days of vaccination was 100% for both doses. The pain induced by injection had little effect on the daily lives of the majority of the recipients.

**Table 3:** Characteristics of receivers with pain following vaccination.

Variables	First-dose vaccination	Second-dose vaccination
Proportion of receivers with pain	286/2,808 (10.19%)	343/2,732 (12.55%)
Sex (male/female)	88/198	102/241
Allergy histories	51 (17.83%)	40 (11.66%)
Medical histories	19 (6.64%)	27 (7.87%)
**Occupation**		
Doctor	77 (26.92%)	83 (24.20%)
Nurse	87 (30.42%)	76 (22.16%)
Technician	38 (13.29%)	26 (7.58%)
Administrator	29 (10.14%)	5 (1.46%)
Researcher	9 (3.15%)	3 (0.87%)
Cleaner	13 (4.55%)	1 (0.29%)
Other	33 (11.54%)	149 (43.44%)
**Pain level**		
1	209 (73.08%)	61 (17.78%)
2	29 (10.14%)	125 (36.44%)
3	21 (7.34%)	100 (29.15%)
4	20 (6.99%)	47 (13.70%)
5	7 (2.45%)	9 (2.62%)
>5	0 (0%)	1 (0.29%)
**Time the pain started**		
Within 5 min after inoculation	33 (11.54%)	35 (10.20%)
Within 5–30 min after inoculation	10 (3.50%)	6 (1.75%)
Within 30 min to 24 h after inoculation	214 (74.83%)	272 (79.30%)
Within 24 to 48 h after inoculation	17 (5.94%)	24 (7.00%)
>48 h after inoculation	12 (4.20%)	6 (1.75%)
**Duration of the pain**		
Less than 30 min	6 (2.10%)	2 (0.58%)
30 min–24 h	89 (31.12%)	73 (21.28%)
24–48 h	82 (28.67%)	128 (37.32%)
48–72 h	62 (21.68%)	79 (23.03%)
More than 72 h	47 (26.05%)	61 (17.78%)
**Body part with the most severe pain**		
Local pain	122 (42.66%)	157 (45.77%)
Upper arm	143 (50.00%)	172 (50.15%)
Other	34 (11.89%)	14 (4.08%)
**Complication**		
Drowsiness	12 (4.20%)	7 (2.04%)
Dizziness	11 (3.85%)	8 (2.33%)
Fatigue	11 (3.85%)	14 (4.08%)
Cold	5 (1.75%)	4 (1.17%)
Fever	4 (1.40%)	2 (0.58%)
Rash	2 (0.70%)	0 (0.00%)
Palpitation	1 (0.35%)	0 (0.00%)
Skin itching	2 (0.70%)	1 (0.29%)
Local induration	4 (1.40%)	5 (1.46%)
Muscle soreness	8 (2.80%)	31 (9.04%)
Night sweat	0 (0.00%)	1 (0.29%)
Other	14 (4.90%)	6 (1.75%)
No symptoms	228 (79.72%)	273 (79.59%)
**Pain relieved (%)**	286 (100%)	343 (100%)

### Prognosis of severe adverse events following COVID-19 vaccination

The AEs that resulted in hospitalization or prolonged hospitalization were classified as severe adverse events (SAEs). There were four cases of SAEs that occurred after the first dose of vaccination and recovered fully with no subsequent discomfort after treatment. The results are shown in **Supplementary Table S3**.

A case of SAE occurred within 48 h after the first dose of vaccination in a 31-year-old woman with no allergy and medical histories. Her main clinical manifestations were fever (37.4°C), local pain (score of 1), skin itching, rash, gastrointestinal reaction, dizziness, headache, fatigue, and dyspnea for more than 72 h. After hospitalization with symptomatic treatment, she fully recovered at follow-up. The second case of SAE occurred in a 56-year-old man with no allergy and medical histories. His symptom was a whole-body rash, the onset time of which was more than 72 h after the first dose of vaccination and lasted for more than 72 h. No other abnormality was observed in further medical tests. Anti-anaphylactic treatment was arranged. The symptom gradually relieved with the skin back to normal. The third case of SAE occurred in a 66-year-old woman who had medical histories of hypertension and cerebral artery stenting. After the vaccination, she had unstable blood pressure. After 12 h, she came back home and then fainted, which led to fractures. She was taken to the hospital by her family and recovered at the time of the report. The last case of SAE occurred in a 37-year-old woman who had medical histories of ovarian and kidney cysts. The onset time of SAE was 24–48 h after vaccination and it lasted for 48–72 h. Her main clinical manifestations were fever (37.8°C), local pain (score of 3), dizziness, headache, fatigue, and acute cystitis. She received anti-infective therapy in the hospital and recovered at the time of the report.

There were two cases of SAEs that occurred after the second dose of vaccination, and they recovered fully with no subsequent discomfort after treatment. The results are shown in **Supplementary Table S4**. The first case of SAE occurred in a 54-year-old woman with no allergy and medical histories. The onset time of SAE after vaccination was greater than 48 h and it lasted over 48 to 72 h. After the vaccination, her index finger knuckles began to twitch and ache, and then she felt pain in the bones and joints all over the body. She went to the hospital for treatment and recovered at the time of the report. The second case of SAE occurred in a 62-year-old woman with no allergy and medical histories. The onset time was 30 min–24 h after vaccination and it lasted for more than 72 h. Her main clinical manifestations were eye pain, blurred vision. She went to the hospital for treatment and recovered at the time of the report.

## Discussion

This study reported the AEs among inactivated COVID-19 vaccine recipients. Data were collected among a total of 2,808 hospital employees and their family members in Wuhan, China, with all of them receiving the first dose of inactivated COVID-19 vaccines from two pharmaceutical companies. Most of the common AEs were mild and tolerable, and the overall incidence of AEs was lower than the data from the safety profile in a phase III clinical trial. The incidence of AEs was higher in the first dose (21.30%, 598) than in the second dose (16.07%, 439). The AEs involved the skin, muscle, respiratory tract, gastrointestinal tract, cardiovascular system, and other tissues and systems. The most common AE was pain at the injection site (first dose: 10.19%, second dose: 12.55%). All the vaccinees with AEs for both doses recovered fully in the end. It was noted that some AEs might cause blood coagulation disorder and bleeding risk. Therefore, ongoing monitoring of AEs after COVID-19 vaccination is essential in evaluating the benefits and risks of each vaccine.

In this study, fewer people received the second dose than the first. Notably, the first injection had more AEs than the second injection. Moreover, the first injection had more severe adverse events than the second injection. Some of the people who received the first dose and had AEs would give up the second dose mainly because they were afraid of other adverse events. The reason why fewer AEs were observed in the second dose could be due to people’s avoidance of the vaccine or the vaccinees’ bodies had already tolerated the vaccine (8).

Pain with an incidence rate of about 10% ranked as the first highest frequent AE, and muscle soreness with an incidence rate of about 2% ranked as the second highest frequent AE. Cold-related symptoms and skin, gastrointestinal tract, cardiovascular, and nervous system disorders were the common AEs. In particular, we identified some scarce but concerning coagulation function disorders with incidence rates of less than 0.3%, such as a bleeding spot on the medial thigh, hematuresis, irregular menstruation, and bloodshot congested eyes.

Moreover, the majority of the recipients (more than 79%) who experienced pain had no other complications and the pain was relieved within 7 days for all of them. The pain induced by the COVID-19 vaccination had a little effect on the daily lives of the recipients. Of note, AEs of pain were more prevalent in female receivers than male receivers. It has been suggested that gender is an important factor in the modulation of pain (9). Men and women differ in their responses to pain, being more variable in women than men. The pain sensitivity increased and many more painful diseases were commonly reported among women (10). Moreover, the second dose was more painful than the first, and the pain occurred later and lasted longer.

All SAEs with incidence rates of less than 0.15% recovered to normal after supportive treatment. The prognosis of SAE was quite well. Both clinical and non-clinical trial data of COVID-19 vaccines have reported that the most common cutaneous reactions are reactions in the local injection site. Dermatologic AEs of COVID-19 vaccines in our study include local induration, local rash, skin itching, and herpes. Dermatologists are suggested to comprehend the landscape of the latest cutaneous reactions to COVID-19 vaccines and tackle vaccinees’ concerns (11–13). It has been reported that systemic inflammatory response could trigger various AEs of COVID-19 vaccines (14). In addition, the systemic inflammatory response could cause modifications in brain physiology, which may be associated with neuropsychiatric symptoms such as neural excitation, hand and face anesthesia, and blurred vision in our study (15).

Of the AEs mentioned above, spike proteins have been reported to play a role in leading to those adverse effects (16). The spike protein of SARS-CoV-2 is responsible to infect human cells. Vaccine makers choose to target this unique protein, multiply the targeted protein, and then theoretically evoke an immune response to prevent it from infecting cells. The spike proteins themselves are responsible for the damage to the coagulation function if they enter the bloodstream (17). Once in the bloodstream, spike proteins attach to specific ACE2 receptors in platelets and cells lining the blood vessels, which can cause platelets to clot (18, 19). Thus, we observed clotting disorders associated with COVID-19 vaccines. Importantly, these spike proteins declined in subsequent weeks, and no spike proteins were detected after the second injection. This is because the individual produces antibodies to remove the antigen from the blood, resulting in an immune response exactly as the vaccine was designed to do (20). Moreover, it is known that vascular endothelial damage is the basis of blood clot formation in blood vessels, which leads to thrombosis and clotting disorders (21). It is also possible that COVID-19 vaccination can induce immune inflammation that targets the endothelium. Therefore, women who had been vaccinated against COVID-19 experienced menstrual disorders.

In summary, most of the common AEs were mild and tolerable, with generally low incidence. The serious adverse event rate was extremely low. The benefits of the coronavirus vaccine outweigh the risks, and all countries continue to promote vaccination.

### Limitations of the study

One of the major limitations of the current study is that we needed to expand the sample size to allow COVID-19 vaccination monitoring to assess vaccine safety. Moreover, different populations need to be studied and sorted out for further research. Also, we were unable to develop a standard questionnaire due to different research purposes to assess COVID-19 vaccine safety in China.

## Conclusions

Most of the common AEs were mild and tolerable, with generally low incidence. Compared with the second dose of vaccination, AEs were more common in the first dose. Of note, coagulation and bleeding disorders may result from AEs. It is essential to continuously monitor AEs after COVID-19 vaccination to evaluate the benefits and risks of each vaccine.

## Data availability statement

The original contributions presented in the study are included in the article/**Supplementary Material**. Further inquiries can be directed to the corresponding authors.

## Ethics statement

The studies involving human participants were reviewed and approved by Union Hospital, Tongji Medical College, Huazhong University of Science and Technology. Written informed consent to participate in this study was provided by the participants’ legal guardian/next of kin.

## Author contributions

MH and WG: literature search, data acquisition, data interpretation, final analysis, and drafting of the manuscript. LL, YY, and QX: data acquisition, interpretation, and supervision. FC: data interpretation and critical revision of the manuscript for important intellectual content. YZ and FZ: literature search, data acquisition, data interpretation, and study conception and design. All authors contributed to the article and approved the submitted version.

## Funding

This work was supported by the Key Research and Development Program of Hubei Province of China (number 2020BCA060) and the National Key Research and Development Program of China (number 2017YFC0909900).

## Conflict of interest

The authors declare that the research was conducted in the absence of any commercial or financial relationships that could be construed as a potential conflict of interest.

## Publisher’s note

All claims expressed in this article are solely those of the authors and do not necessarily represent those of their affiliated organizations, or those of the publisher, the editors and the reviewers. Any product that may be evaluated in this article, or claim that may be made by its manufacturer, is not guaranteed or endorsed by the publisher.
