# Supplementary material for: Safety monitoring in inactivated COVID-19 vaccines by clinical pharmacists from a single center in China
Source: Front Immunol. 2022 Sep 5;13:882919. doi: 10.3389/fimmu.2022.882919 (PMC9483089; doi:10.3389/fimmu.2022.882919)
Supplement: Supplementary file 1 [file DataSheet_1.docx]

**Supplementary Material Presentation**

**Table S1.** The questionnaire for adverse events of inactivated COVID-19 vaccines.

| 1. Basic information | Name / gender / age / id no. / department / professional / mobile phone no. / height (cm) / weight (kg) |
| --- | --- |
| 2. The dose of the vaccine? | ○ The first ○ The second |
| 3. Date of vaccination | _________ |
| 4. Vaccination site | _________ |
| 5. Allergy histories | ○ Food______  ○ Drug______  ○ Vaccine______  ○ Other______  ○ None |
| 6. Past medical histories | _________ |
| 7.Pain score | ○0 ○1 ○2 ○3 ○4 ○5 ○6 ○7 ○8 ○9 ○10 |
| 8. The painful area | ○ None ○_________ |
| 9. A high fever after vaccination? | ○ Normal ○ Fever_________ |
| 10. Dizziness, headache and fatigue? | ○ Yes_________ ○ No |
| 11. The skin adverse reaction | Erythema/urticaria/itching/blister/pimples/plaque/nodules/swelling/other/none |
| 12. The rash | ○ Position_______ ○ Size_____ |
| 13. Other adverse reactions | _______ |
| 14. The time of adverse reactions | ○ Within 5 min after inoculation  ○ Within 5-15 min after inoculation  ○ Within 15-30 min after inoculation  ○ Within 30 min-24 h after inoculation  ○ Within 24 h to 48 h after inoculation  ○ > 48h after inoculation______ |
| 15. Duration of adverse reactions | ○ Less than 30 min  ○ 30 min-24 h  ○ 24 h-48 h  ○ 48 h-72 h  ○ More than 72 h |
| 16. Severity | ○ General ○Serious |
| 17. Treatment measures | ________ |
| 18. The ending | ○ Heal ○Upturn ○ Not recovered ○ Sequelae ○ Unknown |
| 19. Are you sure? | ○ Yes ○ No |

**Table S2.** Univariate analysis of factors associated with AEs in First-dose vaccination group (n=2808).

| Variables | Category | First-dose  vaccination | AEs | OR | 95%CI | P.value |
| --- | --- | --- | --- | --- | --- | --- |
| **Total** |  | 2808 | 598 |  | | |
| **Sex** |  |  |  |  |  |  |
|  | Male  Female | 1357 | 166 | 0.96 | 0.87-0.98 | <0.001 |
|  |  | 1451 | 432 |  |  |  |
| **Age** |  |  |  |  | | |
|  | 18-29 | 895 | 164 | 1.15 | 0.96-1.37 | 0.13 |
|  | 30-39  40-49 | 837 | 155 |  |  |  |
|  |  | 595 | 136 |  |  |  |
|  | 50-59 | 459 | 138 |  |  |  |
|  | 60-70 | 22 | 5 |  |  |  |

**Table S3.** The cases of severe adverse events for the first-dose.

| Age,  years | Sex | Past history | | Onset after  receipt | Duration | Signs and symptoms | Treatment | Outcome |
| --- | --- | --- | --- | --- | --- | --- | --- | --- |
|  |  | Allergies | Previous diseases |  |  |  |  |  |
| 31 | F | No | No | 48h | >72h | Low fever (37.4℃), local pain (1 score), skin itching, rash, gastrointestinal reaction, dizziness, headache, fatigue, and dyspnea | Hospitalize | Recovered |
| 56 | M | No | No | >72h | >72h | The whole body rash | Anti-anaphylactic | Recovered |
| 66 | F | No | Yes, Hypertension, cerebral artery stenting | 12h | >72h | Fainting induced fractures | Inpatient | Recovered |
| 37 | F | No | Yes, Ovarian cysts, kidney cysts | 24-48h | 48h-72h | Fever (37.8℃), local pain (3 score), dizziness, headache, fatigue, and acute cystitis | Anti-infective therapy | Recovered |

Abbreviations: F, female; M, male.

**Table S4.** The cases of severe adverse events for the second-dose.

| Age,  years | Sex | Past history | | Onset after  receipt | Duration | Signs and symptoms | Treatment | Outcome |
| --- | --- | --- | --- | --- | --- | --- | --- | --- |
|  |  | Allergies | Previous diseases |  |  |  |  |  |
| 54 | F | No | No | >48h | 48h-72h | Index finger joint convulsions, bone and joint pain | Hospitalize | Recovered |
| 62 | F | No | No | 30min-24h | >72h | Eye pain, blurred vision, visual object of flying mosquito and mesh shape | Hospitalize | Recovered |

Abbreviations: F, female; M, male.
